# Microbial Diversity in a Hypersaline Sulfate Lake: A Terrestrial Analog of Ancient Mars

**DOI:** 10.3389/fmicb.2017.01819

**Published:** 2017-09-26

**Authors:** Alexandra Pontefract, Ting F. Zhu, Virginia K. Walker, Holli Hepburn, Clarissa Lui, Maria T. Zuber, Gary Ruvkun, Christopher E. Carr

**Affiliations:** ^1^Department of Earth, Atmospheric and Planetary Sciences, Massachusetts Institute of Technology, Cambridge, MA, United States; ^2^Department of Molecular Biology, Massachusetts General Hospital, Boston, MA, United States; ^3^Department of Biology, Queens University, Kingston, ON, Canada; ^4^Department of Genetics, Harvard Medical School, Boston, MA, United States

**Keywords:** mars analog, extremophiles, hypersaline environments, metagenomic, spotted lake, magnesium sulfate

## Abstract

Life can persist under severe osmotic stress and low water activity in hypersaline environments. On Mars, evidence for the past presence of saline bodies of water is prevalent and resulted in the widespread deposition of sulfate and chloride salts. Here we investigate Spotted Lake (British Columbia, Canada), a hypersaline lake with extreme (>3 M) levels of sulfate salts as an exemplar of the conditions thought to be associated with ancient Mars. We provide the first characterization of microbial structure in Spotted Lake sediments through metagenomic sequencing, and report a bacteria-dominated community with abundant Proteobacteria, Firmicutes, and Bacteroidetes, as well as diverse extremophiles. Microbial abundance and functional comparisons reveal similarities to Ace Lake, a meromictic Antarctic lake with anoxic and sulfidic bottom waters. Our analysis suggests that hypersaline-associated species occupy niches characterized foremost by differential abundance of Archaea, uncharacterized Bacteria, and Cyanobacteria. Potential biosignatures in this environment are discussed, specifically the likelihood of a strong sulfur isotopic fractionation record within the sediments due to the presence of sulfate reducing bacteria. With its high sulfate levels and seasonal freeze-thaw cycles, Spotted Lake is an analog for ancient paleolakes on Mars in which sulfate salt deposits may have offered periodically habitable environments, and could have concentrated and preserved organic materials or their biomarkers over geologic time.

## Introduction

Hypersaline environments impose severe stresses on microorganisms, such as high osmotic pressures and potentially low (a_w_ ~0.75) water activities (Grant, 2004). Despite this, life exists over a wide range of salt concentrations in naturally occurring environments with an unexpected level of diversity (Ley et al., 2006). Hypersaline brines have salinities ranging from 35 g/L to more than 400 g/L. Don Juan Pond, a CaCl_2_-dominated Antarctic brine is considered one of the most saline bodies of water on Earth (40–45% by mass; Meyer et al., 1962; Marion, 1997; Dickson et al., 2013), surpassed only by the MgCl_2_-rich Discovery Brine in the Mediterranean, which can reach levels of up to 500 g/L and with the lowest water activity level, *a*_w_ = 0.382, recorded for a brine on Earth (Fox-Powell et al., 2016). Brines can also be highly chaotropic, or membrane destabilizing. Strong chaotropes such as Ca^2+^ and Mg^2+^, when not countered by a suitable kosmotrope (stabilizing ion), prove incredibly hostile to life as evidenced by the apparent lack of viable organisms in both Don Juan Pond and the Discovery Brine. Environments with high levels of kosmotropic sulfate salts however, can sustain life if water activity is sufficiently high (Baldwin, 1996). A saturated MgSO_4_ solution has an *a*_w_ = 0.85 (Ha and Chan, 1999), too low for most bacteria to survive but is habitable to some eukaryotes (Stevenson et al., 2015). At such high salinities, the ionic strength of a solution can also become a problem for microorganisms, where a high charge density can perturb cellular activities (Fox-Powell et al., 2016). Thus, the habitability of a saline environment relies heavily on water activity, a function of the ionic composition and concentration of the brine.

Beyond the Earth, orbital and *in situ* observations of Mars have revealed that extensive water flows, as well as saline and acidic fluids, were once present on the planet's surface (Tosca et al., 2008). Ancient Mars transitioned from wet to dry during the Hesperian (beginning 3.7 Ga), a time of ephemeral lakes, resulting in the widespread deposition of sulfate and chloride salts observed today on the Martian surface (Wanke et al., 2001; Clark et al., 2005; Crisler et al., 2012; Goudge et al., 2016). Magnesium sulfate salts (MgSO_4_•*n*H_2_O) are common on Mars and are distributed globally, with some sediments containing 10−30% sulfate by weight (Vaniman et al., 2004; Gendrin et al., 2005). The presence of hydrated magnesium sulfates within the rim of Columbia Crater is ascribed to the existence of a paleolake, which at times must have been hypersaline in nature (Wray et al., 2011). Targets for future life-detection missions include such salty environments that could have once been habitable, and are relevant today because of their potential to retain water and generate liquid water brines (McEwen et al., 2011; Möhlmann and Thomsen, 2011; Chevrier and Valentin, 2012; Karunatillake et al., 2016).

Most brine environments on Earth contain Cl^−^ as the dominant anion, however, some are rich in SO_4_-bearing salts, such as the Basque Lakes and Hot Lake, which lie within the Thompson Plateau in British Columbia (Jenkins, 1918; Foster et al., 2010). This region, located within the rain shadow of the Coast and Cascade Mountains, has experienced 20 significant glaciations in the last ~1 million years (Church and Ryder, 2010), leaving behind a series of drainage basins with no outlets (endhoreic). A subset of these lakes also have a characteristic “spotted” appearance, including Spotted Lake, which has some of the highest magnesium sulfate concentrations in the world. Such high salt concentrations preserve biosignatures and allow organic compounds, and even entire cells, to be preserved on geologic time scales (e.g., Vreeland et al., 2000; Aubrey et al., 2006). Furthermore, organisms have also been shown to exist in fluid inclusions trapped in rapidly forming salt crystals, and viable isolates have been obtained from inclusions that are on the order of 10^5^ years old (e.g., Mormile et al., 2003; Fendrihan et al., 2006).

Despite the unique geochemical composition of Spotted Lake, the microbial diversity of the environment has not been well studied. Here we describe, for the first time, the biological diversity within the sediments of Spotted Lake in order to identify the types of biosignatures that may be preserved, of relevance to the search for life on Mars.

## Methods

### Field site

**Figure 1:**
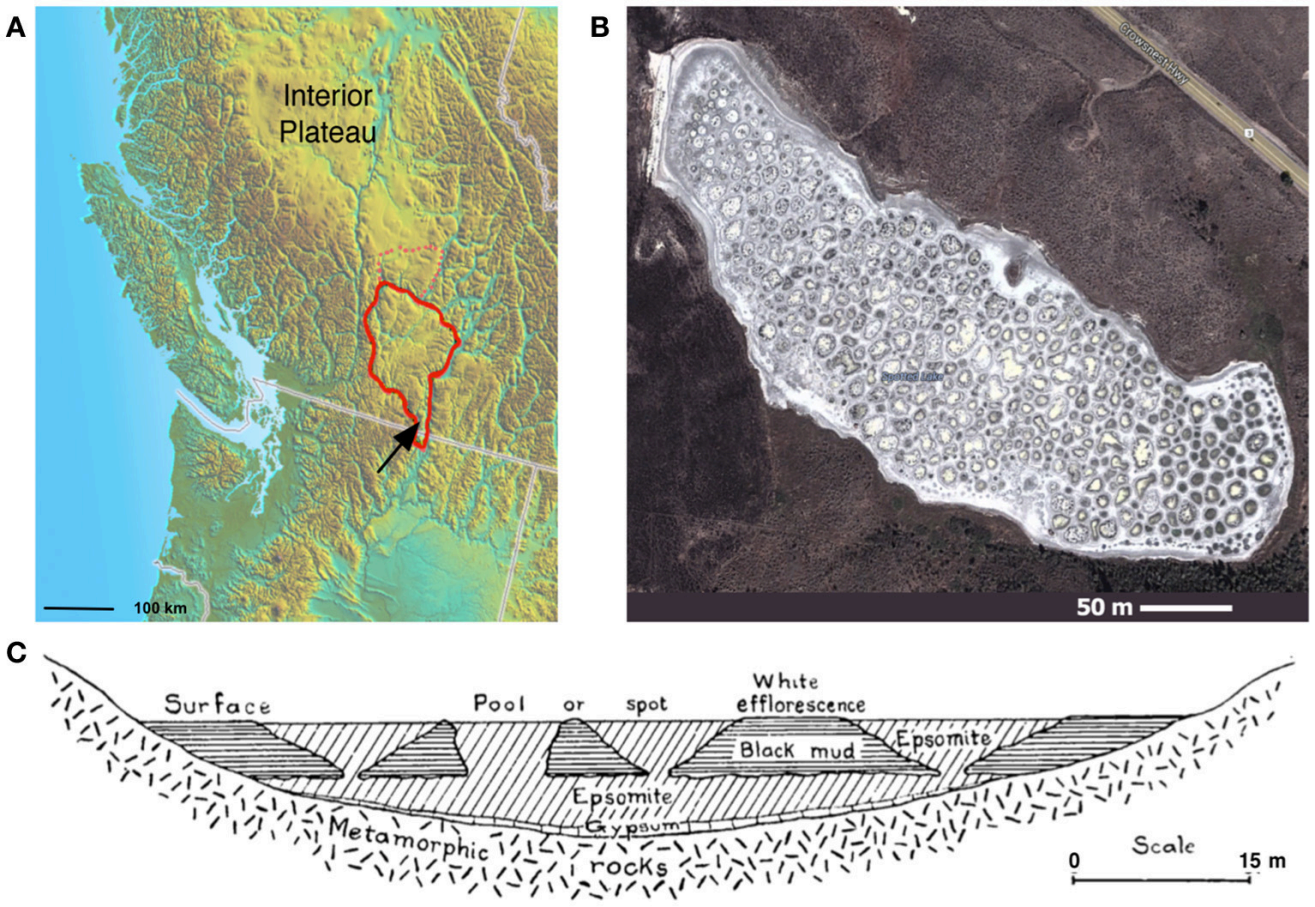
Spotted Lake. **(A)** Spotted Lake (black arrow) is located on the edge of the Thompson Plateau (red line). **(B)** Its hundreds of brine pools are seasonally connected during periods of higher water levels, and separated during periods of low water input and evaporation. **(B)** Imagery © 2014 DigitalGlobe, Map data © 2014 Google. **(C)** During mining of nearby Hot Lake it was discovered that spots represent the bases of inverted cones or cylindrical eposomite masses that connect to a more basal horizontal bed underlain by gypsum. Reprinted from Figure 4 of Jenkins (1918) with permission from the American Journal of Science.

Spotted Lake (Figure 1A, Figure S1) is located in Osoyoos, British Columbia, Canada (49°4′40.86″ N, 119°34′3.01″ W) within Carboniferous to Permian green schist facies rocks, along with dolomites, quartzites, marbles and localized deposits of pyrite and pyrrhotite (Jenkins, 1918). Oxidation of these iron sulfides results in the generation of sulfuric acid and the subsequent weathering of the basin dolomites, yielding high levels of Mg^2+^ and ${\text{SO}}_{4}^{2-}$ that concentrate in the endorheic lake. As a result, Spotted Lake is rich in magnesium and sodium sulfate salts, and with a slightly alkaline pH. Due to low levels of precipitation in this region, summer evaporation leads to the formation of individual brine pools (Figure 1B), which are separated by mud mounds (Figure 1C) and surficial salt crusts (Jenkins, 1918; Cannon et al., 2012). Samples from Spotted Lake were collected in October 2010 (Table 1); in total, 4 individual ponds were surveyed and samples including water and sediment (top 5–10 cm) were aseptically collected in sterile containers. Water samples were collected first (without disturbing the sediment) in autoclaved plastic sterilization units with samples immediately sealed, and subsequently analyzed for pH and ion concentrations following Wilson et al. (2012). Water activity was measured in triplicate from two brine pools in the laboratory using an AquaLab Dew point activity meter 4TE, and on two sediment samples, at a temperature of 25°C. Sediments (45–100 g) from each pond were collected in 50 mL plastic conical tubes and immediately after collection were transferred to glass test tubes, which were sealed with rubber stoppers, purged with nitrogen, and sealed with a crimped metal band before being frozen at −20°C. All tubes were placed in a cooler with freezer packs for shipment, and stored at −80°C upon arrival. Soil samples from each pond were sent for inductively coupled plasma mass spectrometry (ICP MS) analysis, Bureau Veritas, Canada.

**Table 1:** Geochemistry of Spotted Lake water samples.

**Major cations**	**Concentration (mg/L)**	**Molarity (mM)**
Mg	51,400	2,115
Na	42,600	1,835
K	3,010	77
Ca	214	5
**Major anions**	**Concentration (mg/L)**	**Molarity (mM)**
SO_4_	271,000	2,821
Cl^−^	2,700	76
**Other**	**Concentration (mg/L)**	
Hardness	212,000	
Salinity	370,999	
Water activity	0.98	
Si	8.2	

*Values represent an average of four pools*.

### Microscopy

For *in situ* imaging of the environment, soil samples were fixed in glutaraldehyde, dehydrated in ethanol and critical-point dried to preserve cell structure using a Tousimis Auto Samdri 815 Series A Critical Point Dryer following Dykstra and Reuss (2003). Samples were then mounted, carbon coated and imaged using a Zeiss Merlin High-resolution Scanning Electron Microscope (SEM) at 1 kV. Soil samples were also imaged to assess viability: soil was stained using Live/Dead Baclight Bacterial Viability Kit (Life Technologies; now Thermo-Fisher Scientific, Waltham, MA) and then imaged on a Zeiss ApoTome 2.

### DNA extraction and sequencing

DNA extraction was performed utilizing both a high-input process (MoBio) and a low-input process (Zymo) to explore differences in acquired sequencing data due to extraction protocols (Figure S3). (1) High input method: Genomic DNA (gDNA) was extracted using the MoBio Powersoil DNA isolation kit (Carlsbad, CA), and concentrated with Zymo Research Genomic DNA Clean and Concentrate. Gel electrophoresis and a NanoDrop Spectrophotometer (Thermo Scientific) were used to assess gDNA quality and concentration, respectively. (2) Low input method: gDNA extraction was performed (0.25 g from each of the four samples) using Zymo Research Soil Microbe DNA MicroPrep; eluted gDNA was further subjected to whole genome amplification using phi-29 (GE Healthcare Illustra Ready-To-Go GenomiPhi V3) to produce enough DNA for library construction.

The Ion Torrent PGM system (Rothberg et al., 2011), Ion Xpress Fragment Library and Ion Xpress Template kits were purchased from Ion Torrent Systems (Guilford, CT). Sequencing, library construction and template preparation were performed according to the 200 bp Ion Xpress Fragment Library and Template Preparation protocols, and libraries S1–S4 (high input), and Z1–Z4 (low input) for sediment samples 1–4 were constructed. A single sequencing run was then performed (Table S1) on a 316 chip using 500 flows (equivalent to 125 cycles).

### Metagenomic analysis

Raw sequencing reads were analyzed with Phylosift v1.0.1 (Darling et al., 2014) using default parameters, which included quality trimming of FASTQ data. Microbial community structure was assessed directly from sequencing reads through comparison to reference sequences (37 near-universal single-copy genes, 16S and 18S ribosomal genes, mitochondrial genes, eukaryotic-specific genes, and hundreds of virus-specific genes). Raw reads were also submitted to MG-RAST to confirm Phylosift results, and also for further 16S rRNA phylogenetic and protein functional analyses (Meyer et al., 2008; Glass et al., 2010). For MG-RAST, the default quality control options for quality trimming, dereplication, and screening for common contaminants were used.

In order to compare Spotted Lake metagenomes (Table S2) with previously-analyzed metagenomes, keyword searches for salt-associated metagenomes in addition to a few other sets such as air (in order to include data representing exogenous environmental seeding) were conducted. The resulting list was filtered to exclude virus-specific datasets, contigs/assemblies, and datasets associated with a specific organism, giving a final list of MG-RAST metagenomes for comparison (Table S3). For each of these metagenomes a lowest common ancestor (LCA) analysis was performed at the species level using MG-RAST with the default settings (max *e*-value 10^−5^, min identity cutoff 60%, min alignment length cutoff 15 bp). An abundance matrix was constructed with one row per metagenome and one column for each unique taxonomic key across all metagenomes. The unique set of taxonomic keys was generated at each taxonomic level and a separate PCA analysis was done for each taxonomic level from domain to species (see Supplementary Material, pg. 2).

## Results

### Geochemistry

**Figure 2:**
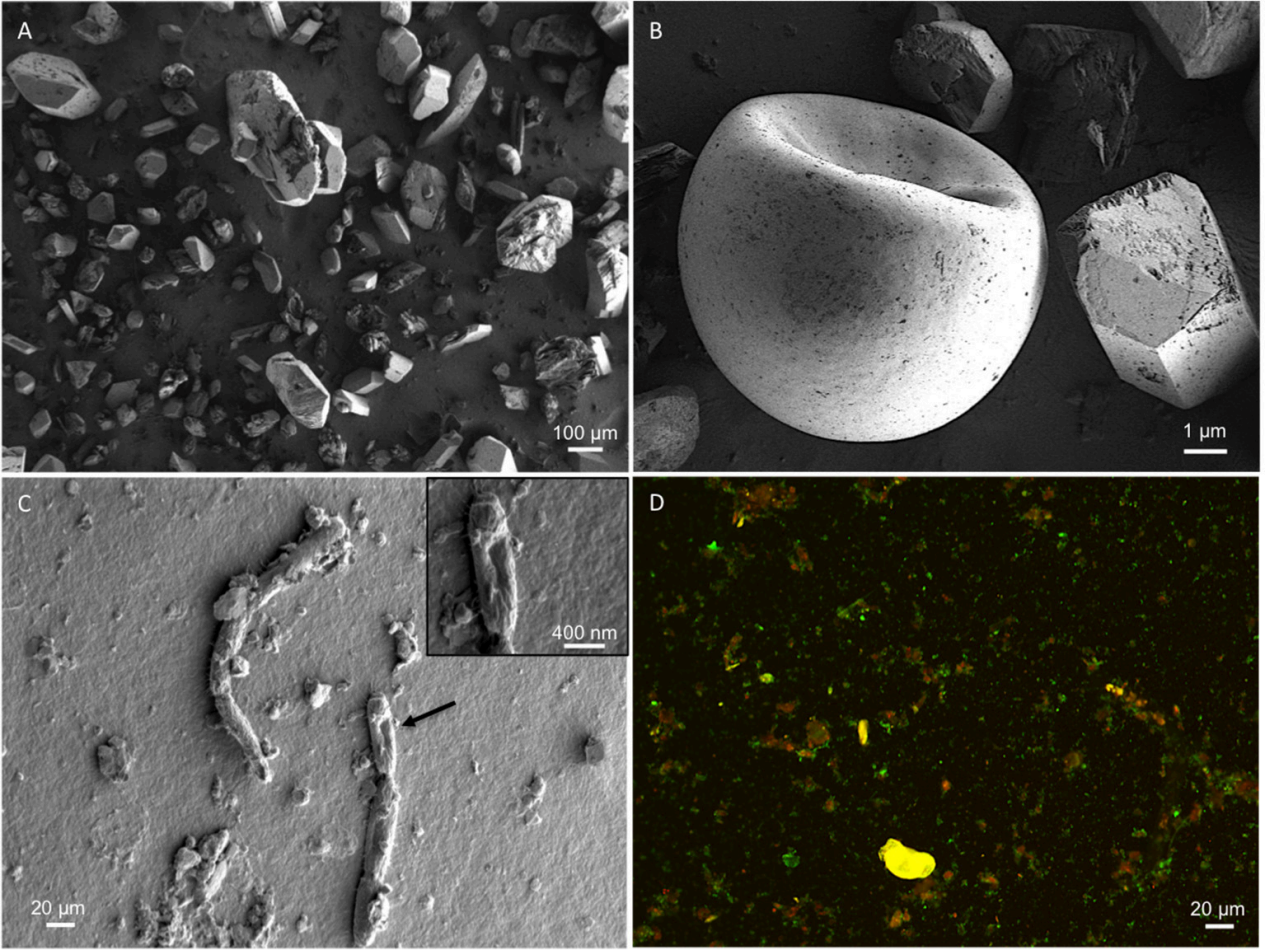
SEM micrographs. Representative micrographs show **(A)** the mineral substrate consisting of sulfate salts; **(B)** a brine shrimp egg (one of many hundreds seen in other micrographs), revealing the presence of higher-order biology in the system; **(C)** presence of *in situ* microbes within the soil samples; **(D)** Sediment-derived microbes, visualized with SYTO 9 and propidium iodide. Original micrographs are available in the Supplementary Material, Figures S4–S8.

Water pH ranged from 8.0 to 8.3, and *a*_*w*_ was 0.98 for the water column, and ranged from 0.96 to 0.99 within the sediment. Water chemistry measurements revealed brine compositions consisting of SO_4_ (2.8 M), Mg (2.1 M), and Na (1.9 M), with minor contributions from K and Cl (Table 1), nearly identical to the 1933 historical measurements (McKay, 1935). Total salinity was measured at 37.1% with an approximate molar ratio of MgSO_4_:Na_2_SO_4_ of 20:9 consistent with previous identification (Cannon et al., 2012) of minerals including epsomite (MgSO_4_•7H_2_O) and mixed Mg-Na salts in various hydration states (Figure 2A), e.g., blöedite, konyaite. ICP-MS (Table 2) revealed very low silica amounts, <8%, and CaO levels ranging from 17 to 25%. The percentage total for the major oxides ranged from 62 to 66%, which was representative of the fact that a large component of the sediment was comprised of salt and not detectable using the methodology employed. When subtracted from the ideal total of 100%, the remaining ~33−37% was inferred to be sulfate salt, corresponding with the sulfate concentrations of the water column. Total organic carbon was low, ranging from 1 to 3%.

**Table 2:** ICP-MS results for major oxide, total organic carbon, total sulfur composition, and sulfate from the sediment of Spotted Lake.

**Sample**	**Mineral Composition(% Weight)**															
	**SiO_2_**	**Al_2_O_3_**	**Fe_2_O_3_**	**MgO**	**CaO**	**Na_2_O**	**K_2_O**	**TiO_2_**	**P_2_O_5_**	**MnO**	**Cr_2_O_3_**	**LOI**	^*^Total	^*^SO_4_	**TOC**	**TOS**
SL_1	6.13	1.27	0.64	5.12	17.56	8.42	0.33	0.08	0.06	0.02	<0.002	22.8	62.59	37.41	3.1	15.94
SL_2	7.96	1.72	0.85	4.32	25.01	3.43	0.37	0.12	0.08	0.02	<0.002	20.7	64.78	35.22	2.37	15.52
SL_3	3.23	0.7	0.33	4.32	24.1	3.02	0.25	0.05	0.03	0.01	<0.002	29.3	65.55	34.45	0.99	15.5
SL_4	6.08	1.28	0.68	3.78	25.14	2.03	0.35	0.09	0.05	0.02	<0.002	27	66.76	33.24	1.12	15.36

* *Sulfate could not be directly measured by this method and thus is inferred by subtracting the total major oxide percentages from 100. The calculated levels are in keeping with concentrations of sulfate salts measured in the water column, and account for the low Total levels acquired from ICP-MS*.

### Metacommunity

**Figure 3:**
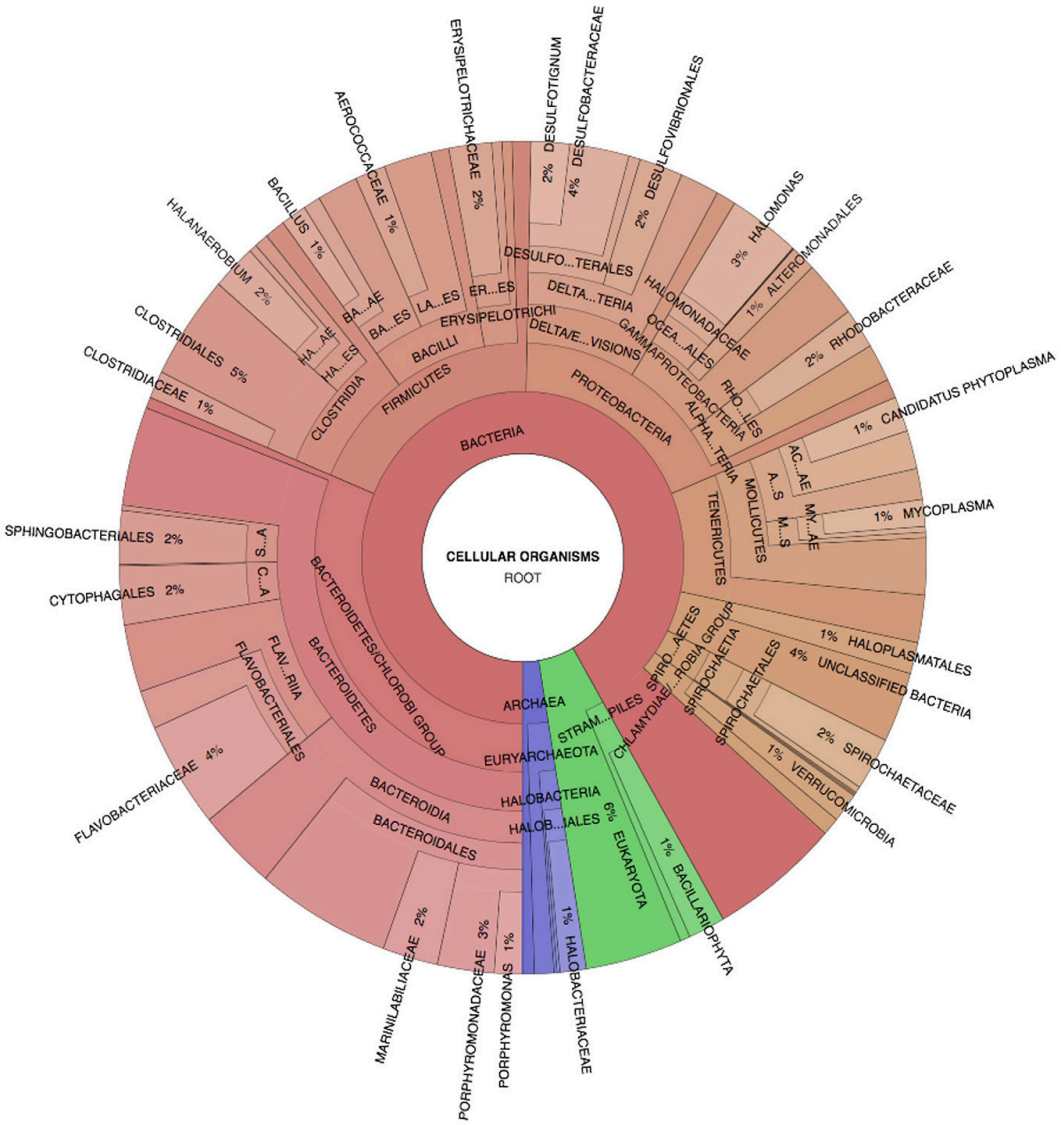
Spotted Lake sediment assemblages. Community composition based on Phylosift-estimated abundance within the representative pooled, high-input sample S2, Legend: Eukaryota (blue), Archaea (green), and Bacteria (Red/Orange), with lighter shades indicating lower taxonomic rank (toward species level resolution).

**Figure 4:**
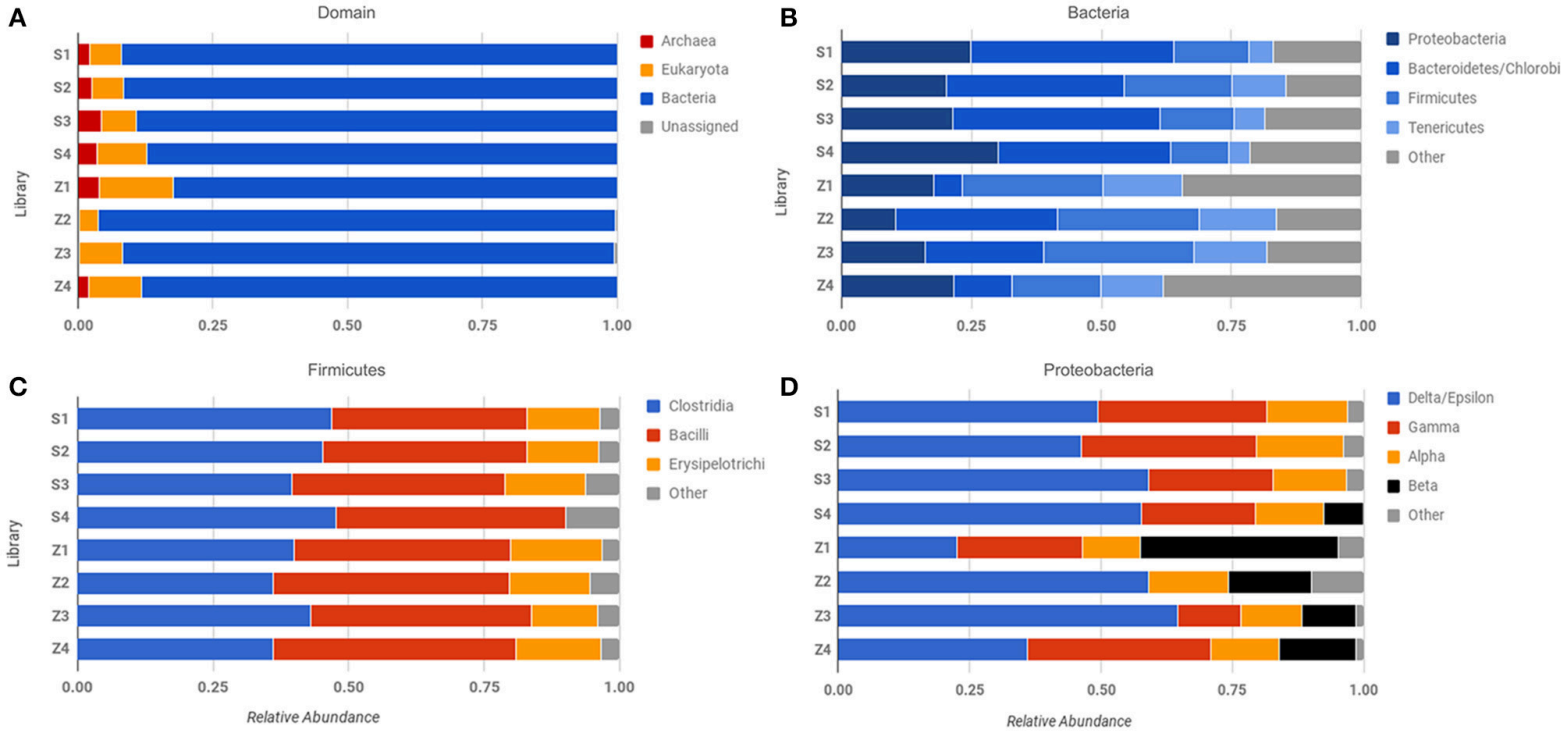
Comparison of abundance across different sequencing libraries and samples. Phylosift-estimated abundance at the level of **(A)** Domain, **(B)** Bacteria, **(C)** Firmicutes, and **(D)** Proteobacteria. Row labels correspond to specific sequencing libraries (see text and Table S1).

Spotted Lake was found to be host to a diverse set of organisms, from macroscopic brine shrimp (Figure 2B) to a wide range of bacteria (Figure 2C). Imaging of the community through Live/Dead staining revealed a viable population (Figure 2D). The abundance assessment of the S1–S4 and Z1–Z4 libraries (representing “high input” vs. “low input” modalities) pooled, revealed respectively 90 and 88% Bacteria, 10 and 4% Eukarya, and 3 and 3% Archaea (Figure 3). The S1–S4 and Z1–Z4 libraries varied from each other (Figures 4A–D), especially at the sub-domain level. Within the high input libraries, dominant bacterial phyla included Bacteroidetes (33.2%), Proteobacteria (21.4%), and Firmicutes (14.1%). Alternatively, the low-input Z1–Z4 library had a dominant contribution from the Firmicutes (21.3%) only. The highest abundance proteobacterial classes in S1–S4 were δ/ε-proteobacteria (11.2%), γ-proteobacteria (6.1%), and α-proteobacteria (3.2%). While highly diverse at the family to species level, halophiles were well represented, including the genus *Halomonas*, previously found in hypersaline (NaCl) environments, representing 3% of γ-proteobacterial sequences, and the family Rhodobacteraceae (9%), members of which are common in seawater, where they play a key role in marine carbon cycling (Pujalte et al., 2014). Also present were sulfate reducers of the family Desulfobacteraceae (46%), largely comprised of organisms most closely related to the genus *Desulfotignum*, an obligate anaerobe capable of both chemoorganotrophy and chemolithotrophy (Kuever et al., 2001). Sequences belonging to the classes of bacteria lacking a cell wall were also represented: Mollicutes (8%) and Haloplasmatales (1%), the former of which is typically a parasite of eukaryotes (Skennerton et al., 2016), and the latter which is found only in hypersaline environments (Antunes et al., 2008).

Our results underscore the potential for bias in community characterization between “low-input” and “high-input” methodologies, with implications for future Martian expeditions. Not all low-input samples yielded evidence of Archaeal sequences (Figure 4A), but consistently demonstrated the presence of β-proteobacteria, which were largely absent in the “high-input” libraries (Figure 4D). Utilizing small sample sizes can result in an over- or underrepresentation of species due to the location of the subsample within the larger context, especially where physicochemical boundaries are present, thus care must be taken to extract multiple sub-samples that adequately represent the larger sample of interest. Beyond the issues noted with small sample sizes, whole genome amplification with phi-29 polymerase does result in a preference toward A+T rich genomes (Yilmaz et al., 2010), which may account for the dominance of the Firmicutes in these samples.

**Figure 5:**
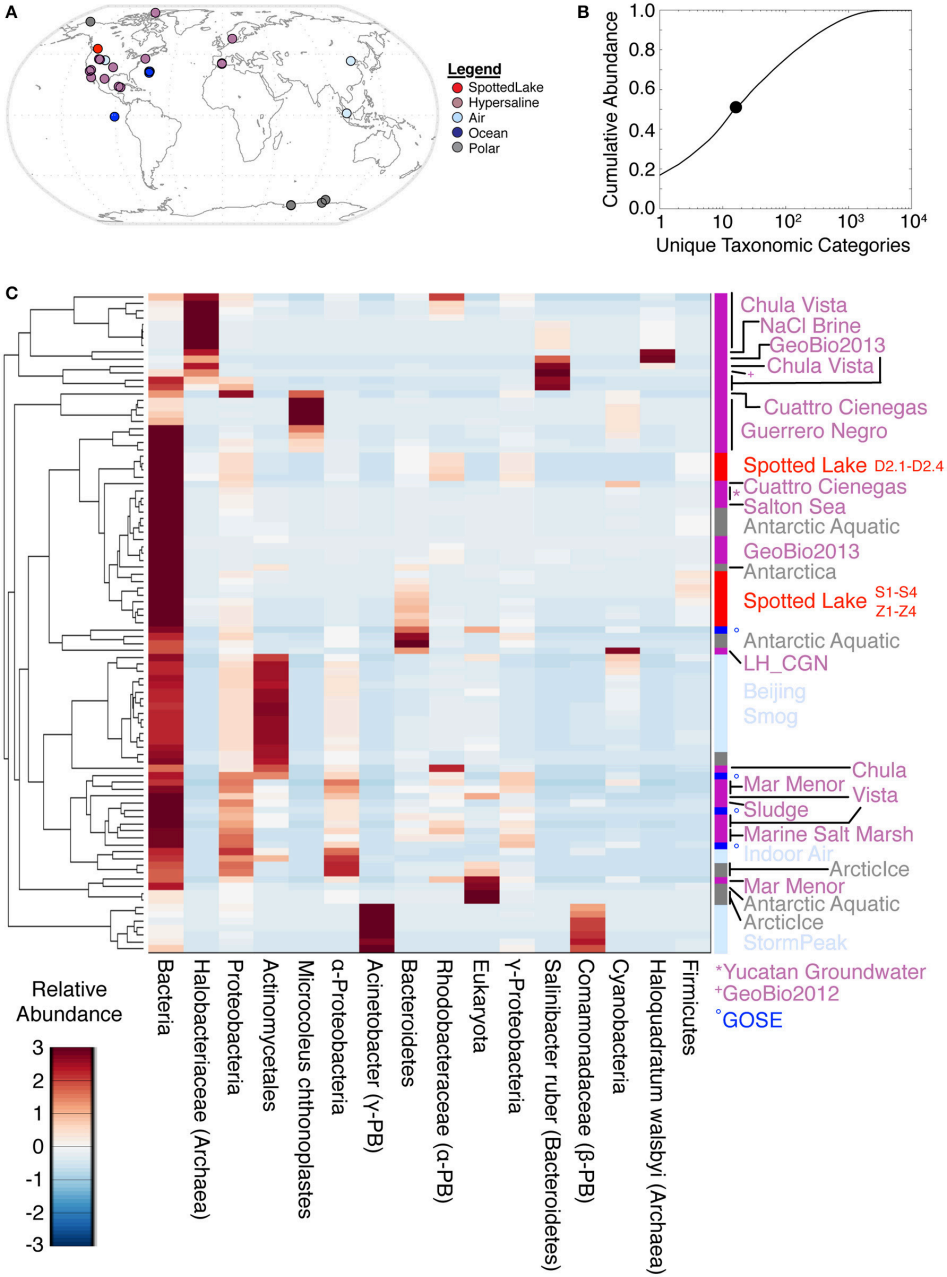
Lowest common ancestor (LCA) abundance comparison of metagenomes. **(A)** Comparator metagenomes (86) were selected from hypersaline, air, polar, and ocean environments representing 20 different sites or projects (Table S3). **(B)** The 16 taxonomic categories of highest abundance capture 50.4% of the cumulative abundance. **(C)** Metagenomes clustered by similarity based on the 16 most abundant taxonomic categories.

Analyses of alpha diversity were abundance weighted and calculated using the Shannon Diversity Index (*H'*). *H'* ranged from 599 to 704, with an average of 666 for high-input samples, and 680 to 1,122, with an average of 802, for low-input samples. To compare species abundance across all metagenomes (Figure 5A), a lowest common ancestor (LCA) analysis was performed, using classification down to the species level. This yielded hits in 17,433 unique taxonomic categories, though half of the abundance was captured by only 16 taxonomic categories (Figure 5B). Assessment of the metagenomic datasets using these highly abundant taxonomic categories revealed some similarities between Spotted Lake and both hypersaline and Antarctic environments (Figure 5C).

**Figure 6:**
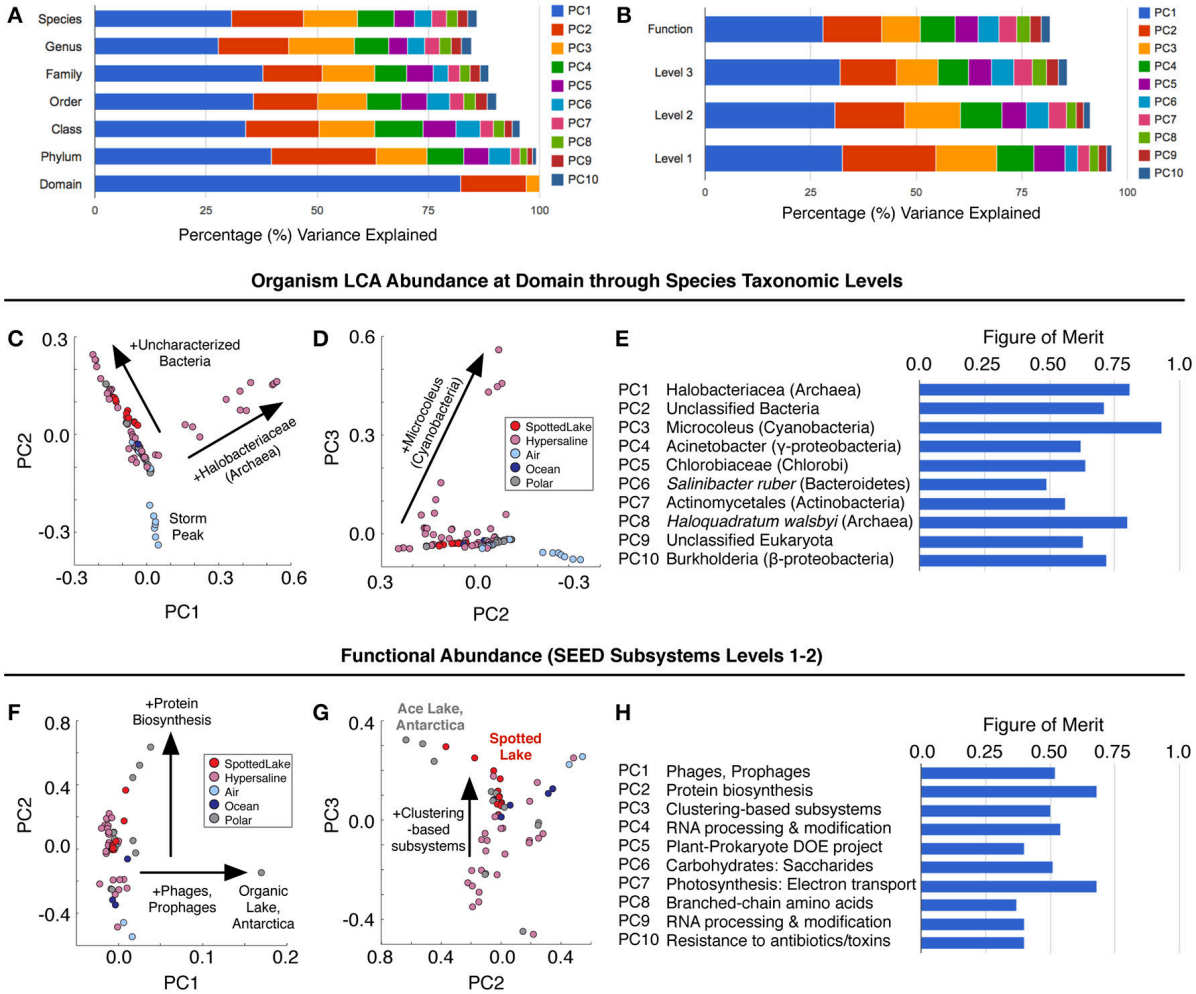
Variation in abundance and function across metagenomes. **(A)** Principal component analysis (PCA) of Lowest Common Ancestor (LCA) -derived abundance at different taxonomic levels. **(B)** PCA of SEED subsystem classification of protein sequences at different functional levels. **(C–E)** Principal components scores based on LCA abundance are associated with specific taxonomic signatures (suggesting a way to classify hypersaline environments). The figure of merit **(E)** indicates the precision of the correlation between a PC score and a specific taxonomic signature (see text and methods for details). **(F–H)** Function-based PCA scores **(F–G)** reveal different variation patterns from abundance-based PCA scores, and lower figures of merit, implying a reduced correlation between PCs and specific functions.

Principal components analysis (PCA) on the LCA abundance data (Figure 6A) revealed a similar pattern of explanatory power at each level of taxonomic depth: the first three principal components (PCs) explained 58–62% of the taxonomic variation from class to species. Thus, we focused on the species level analysis (Figures 6C–E), which provides the most specific taxonomic classification at a cost of slightly reduced explained variance for higher-order PCs.

Abundance analysis revealed that the hypersaline environments studied could be distinguished by three main taxonomic signatures: (archaeal) *Halobacteria* (associated with the first principal component, or PC1), unclassified bacterial sequences (PC2), and Cyanobacteria (PC3). These three main directions of variation represent the observed combinations of taxonomic abundances: For example, metagenomes from Chula Vista water samples have moderate to high PC1 scores and are dominated by Halobacteriaceae (Figure 5C). Occupying another niche are microbial mat samples from Guerrero Negro, which have low PC1 and range from low to high PC3 scores that are inversely related to PC2, so that high levels of *Microcoleus* are associated with low levels of uncharacterized bacteria, and vice versa (Figure 5D); see Table S3 for metagenomic datasets.

To describe the strength between these taxonomic signatures and each PC, a figure of merit (FoM) was defined, which is a score ranging from 0 to 1, where 0 indicates no association with a PC score, and 1 indicates perfect correlation (a PC is uniquely associated with variation of the specified taxonomic signature). Higher-order PCs are similarly associated with specific taxonomic signatures (Figure 6E) with high figures of merit. These associations corroborate the patterns of abundance visible in Figure 5C. A similar analysis was also carried out using SEED Subsystems database (Overbeek et al., 2005) for functional analysis of protein sequences (Figure 6B). Functional analysis (Figures 6F,G) revealed a tighter grouping of hypersaline environments than found based on abundance (Figures 6C,D). Functional variation was associated with phages (PC1), protein biosynthesis (PC2), and clustering-based subsystems (PC3). Metagenomes derived from Ace Lake, Antarctica, clustered in a similar fashion to Spotted Lake along PC2 and PC3 (Figure 6G), in part due to high abundance of clustering-based subsystems, a label given to genes with unknown function but presumed functional coupling (appearance together within multiple genomes, such as within an operon). The function-based PCs were less associated with specific functions than in the abundance analysis (Figure 6H; figure of merit 0.44 ± 0.18 vs. 0.69 ± 0.12 for abundance analysis, mean ± s.d.).

## Discussion

The high concentration of sulfates within Spotted Lake makes this one of the most hypersaline environments in the world—yet microbial life is both abundant and diverse, indicating that hypersalinity in and of itself is not a barrier to microorganisms. Rather, habitability is likely more dependent on the water activity and chaotropicity of the brine, controlled by the ionic strength and composition of the solution. Community abundance in the Spotted Lake sediments likely reflect both their geochemical setting (anaerobic organisms such as Firmicutes and Bacteroidetes, sulfate reducers, halophiles) and the integration of exogenously-delivered organisms with microbes transported via aerosols and precipitation on a global scale, seeding populations of facultative anaerobes and aerotolerant organisms. Because salt and other ion levels fluctuate dramatically throughout the year in the water column (Figure S2) with impact on precipitation rates and pore water concentrations in the sediment, we expect that community composition in Spotted Lake sediments may vary seasonally, similar to the archaeal abundances in Lake Tyrrell, Victoria, Australia (Podell et al., 2014) or the relative abundance of Proteobacteria and Cyanobacteria in the waters of nearby Hot Lake, Washington (Crisler et al., 2012; Lindemann et al., 2013).

In comparison to other hypersaline environments, Spotted Lake is notable for its high sequence diversity, reflected in the high abundance of “unclassified bacteria” present, as well as its low levels of Archaea (*Halobacteria*). The latter are aerobes that grow optimally at mesophilic temperatures, and likely their low abundance reflected the anoxic nature of our sediment samples and the temperate environment. The lack of Cyanobacteria, and indeed any phototroph, in our samples was surprising given that they thrive in the water of nearby Hot Lake (Lindemann et al., 2013; Kilmer et al., 2014), and have been observed within the salt crusts of Spotted Lake (Cannon, *pers. comm*.). It is possible that in Spotted Lake these values are indicative of low light levels in the water column, where the situation of the lake (surrounded by hills) limits the amount of direct sunlight received, which is then further mitigated by a large amount of absorbance and scattering of any incident light by the overlying salt layer. Further exploration of the water column will be required to address the absence of detrital eDNA from phototrophs. The tighter grouping of hypersaline environments based on functional analyses emphasizes the number of uncharacterized, but connected genes within these systems. For example, Spotted Lake's taxonomic and functional similarities to Ace Lake, Antarctica may reflect their similar sulfidic, anaerobic environments, which have nearly identical pH (Rankin et al., 1999), though salinity within Spotted Lake is higher by an order of magnitude. However, it is also clear that both extreme environments harbor functionally connected but unknown genes, and thus largely harbor uncharacterized microbes. The extent to which many sequencing reads could not be assigned at the taxonomic levels of family, genus, and species is consistent with the presence of additional uncharacterized extremophiles.

We identified little evidence of representatives from the viral domain of life (phages, eukaryotic viruses, and virophages). In marine environments, phages typically outnumber cells by a factor of 5–25 and represent 5% of biomass (Suttle, 2007). Even in oligotrophic deep sea sediments, characterized by low cell counts and extremely low metabolic rates and turnover, phage to cell ratios can be similar or even higher (Engelhardt et al., 2014), consistent with both active microbial metabolism and viral-induced death; scientists have also found evidence of virophages within Ace Lake, which pray on viruses that infect phototrophic algae (Yau et al., 2011), regulating host-virus interactions and influencing overall carbon flux in the system. If viruses are present in Spotted Lake sediments, we failed to detect them either due to a dominance of RNA viruses to the exclusion of DNA viruses, loss of DNA viruses during extraction despite mechanical disruption techniques known to generally yield viral DNA, or any viral sequences fell within the so-called “dark matter of sequence space” for which we lack suitable marker or reference sequences. We did however, identify sequences belonging to the phylum Tenericutes (class Mollicutes) that lack cell walls and are typically parasites of Eukaryotic organisms (Skennerton et al., 2016). Many members of Mollicutes have been identified in hypersaline environments, though not necessarily associated with a host (Skennerton et al., 2016). Our Mollicute sequences did not relate to any known halophilic organisms, and instead were dominated by the Acholeplasmataceae, which are facultative anaerobes and infectious agents of plants (Stephens et al., 1983). A small portion (1%) of our reads belonged to the class Haloplasmatales, which also lacks a cell wall and is an anaerobic, denitrifying bacterium, unique to hypersaline environments (Antunes et al., 2008, 2011)

Approximately 8% of our Bacterial reads belonged to anaerobic sulfate reducers (Desulfobacterales), with 22% of those belonging to the genus *Desulfotignum*, a group of chemoorganotrophs/chemoautotrophs capable of using aromatic compounds as carbon sources or electron donors. In the absence of organic carbon, these organisms are also capable of chemolithoautotrophy, utilizing H_2_ as the electron donor, with sulfate, sulfite and thiosulfate all serving as terminal electron acceptors (Kuever et al., 2001). The abundance of sulfate reducers in this system, and the versatility with which some of them can conduct their metabolisms, indicates that the production and preservation of sulfides within this system is likely quite prevalent, similar to Ace Lake (Burton and Barker, 1979), Lake Lisan (Torfstein et al., 2005), and Mono Lake (Stam et al., 2010). In Mono Lake, the low isotope fractionations at low rates of sulfate reduction were thought to be characteristic of halophilic sulfate reducers (Stam et al., 2010). However, Wing and Halevy (2014) suggested that when conditions of low metabolic activity are coupled with high concentrations of environmental sulfate, the kinetic effect of sulfur isotope fractionation is exacerbated, resulting in highly negative values. Thus, it is possible that a strong isotopic signature may exist within the sediments of Spotted Lake; further study is ongoing.

### Astrobiological implications

Hypersaline environments have been shown to preserve biological material on timescales exceeding that of non-saline systems, thus, the presence of paleo-sulfate lakes on Mars is of particular interest for exobiology, as these deposits may retain evidence of previous microbial habitation on the planet. The biological characterization of Spotted Lake is important in order to further delineate the limits to microbial growth in hypersaline environments: do such conditions on present-day or ancient Mars represent potentially habitable, versus merely organic-preserving, environments? On Mars today, climate is mainly regulated by its obliquity (Mischna et al., 2013), which in recent epochs has varied with a 124,000-year cycle. This cycle alternatively stabilizes and destabilizes ground ice in non-polar regions of Mars, which could alter the probability or frequency of near-surface liquid water brines. While MgSO_4_ has minimal (−3.6°C) freezing point depression, it can undergo supersaturation allowing for an additional (4–6°C) cooling below this eutectic without precipitation, and at some concentrations can be cooled up to 15°C below the eutectic before becoming solid (Toner et al., 2014). Freezing and salt precipitation events as well as their associated latent heat may help buffer these deposits against low Martian temperatures and diurnal fluctuations. It is plausible that salt precipitation and drying-out events could also result in the entrapment of high molarity fluids that still may maintain transient water activity levels high enough to support microbial activity. Any organisms in such brines would have to cope with osmotic stress, desiccation, and freeze-thaw stresses, as well as cosmic irradiation if within 1–2 m of the surface.

## Conclusions

We have shown that Spotted Lake sediments are inhabited by extremely diverse, mostly anaerobic organisms, with low levels of Archaea and a near absence of detected DNA viruses. This community composition reflects both exogenous delivery of organisms and adaptation to the geochemical environment: the significant presence of anaerobes and extremophiles undoubtedly reflects the capacity of microbes to grow and divide in this extreme environment, characterized by sulfate levels near or at saturation, desiccation, and freeze-thaw stresses. Moreover, analysis of the Spotted Lake genomes in comparison with other hypersaline environments points toward a group of functional genes associated with these brine conditions that have yet to be characterized. Analogous sulfate-rich closed-basin paleolakes on Mars would represent excellent locations to search for preserved organic material and associated biomarkers, which would be concentrated through evaporative processes, entombed in sulfates, and preserved within lake-bed deposits, conserving signatures of ancient life that could persist over geologic time.

## Author contributions

AP and CC coordinated and managed the study. VW obtained the samples. VW and AP carried out the geochemical analyses. CC and TZ designed the experiment and CL, TZ, and HR processed the samples. HR performed the sequencing. AP, and CC analyzed the data and wrote the manuscript text. MZ and GR advised on the study and analysis. All authors edited and reviewed the manuscript.

### Conflict of interest statement

The authors declare that the research was conducted in the absence of any commercial or financial relationships that could be construed as a potential conflict of interest.
