# Supplementary figures and images for: Microbial Diversity in a Hypersaline Sulfate Lake: A Terrestrial Analog of Ancient Mars
Source: Front Microbiol. 2017 Sep 26;8:1819. doi: 10.3389/fmicb.2017.01819 (PMC5623196; doi:10.3389/fmicb.2017.01819)

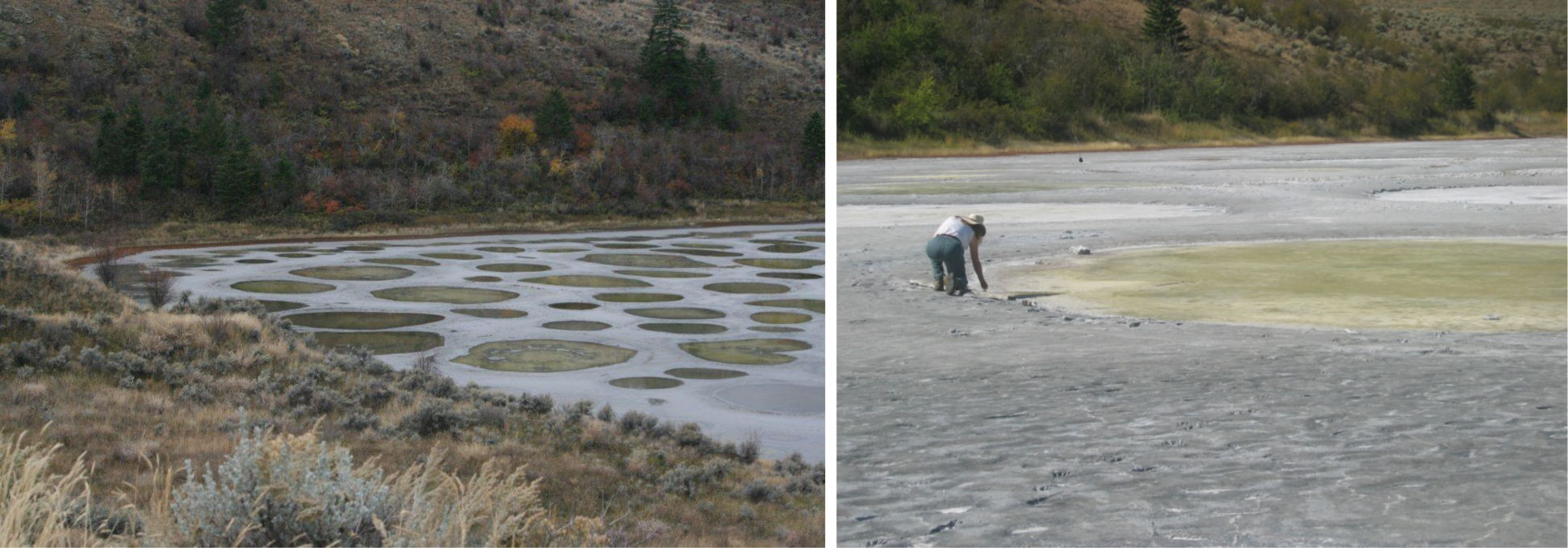

Supplement: Supplementary file 1 [file Image1.TIFF]

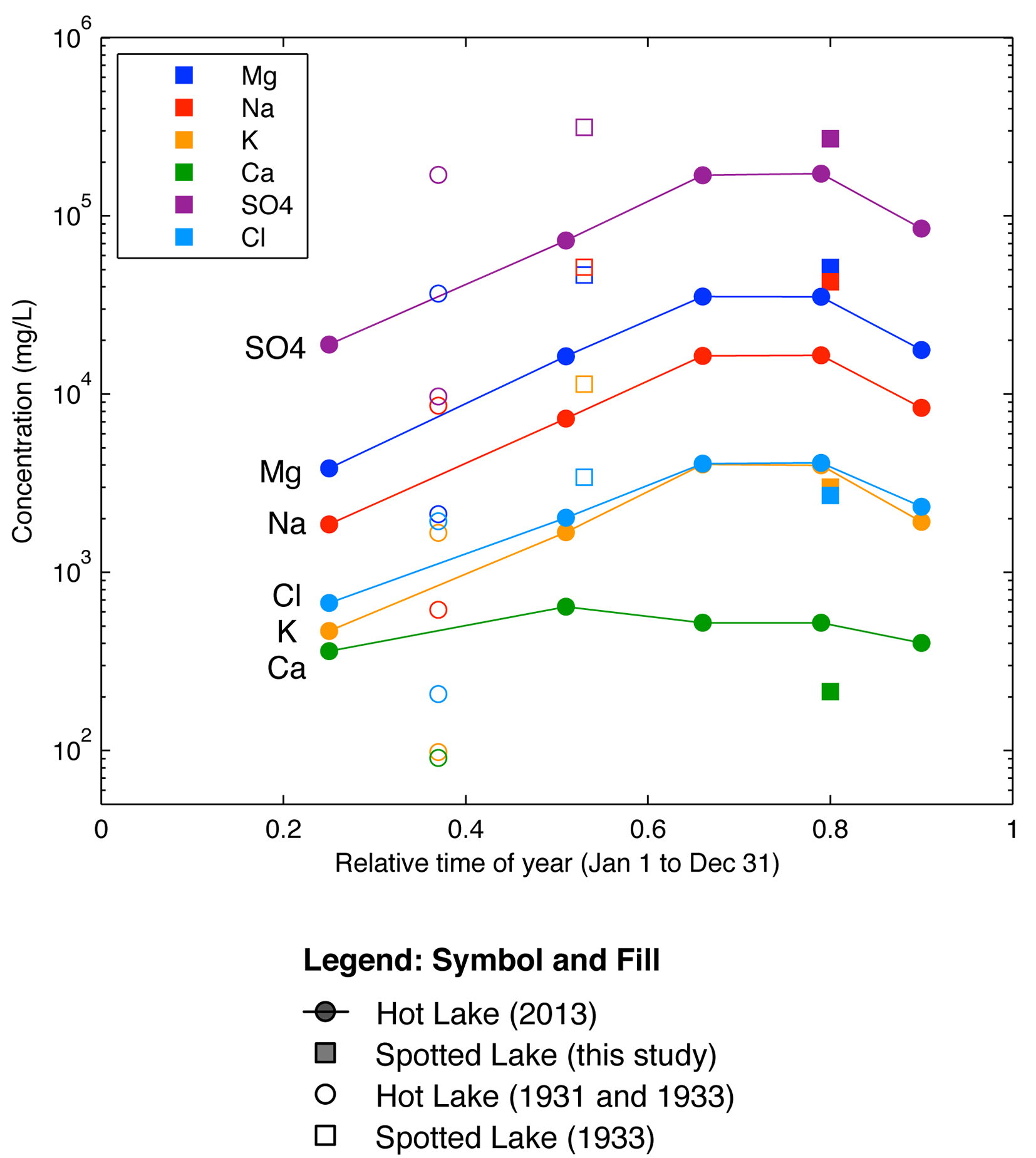

Supplement: Supplementary file 2 [file Image2.TIFF]

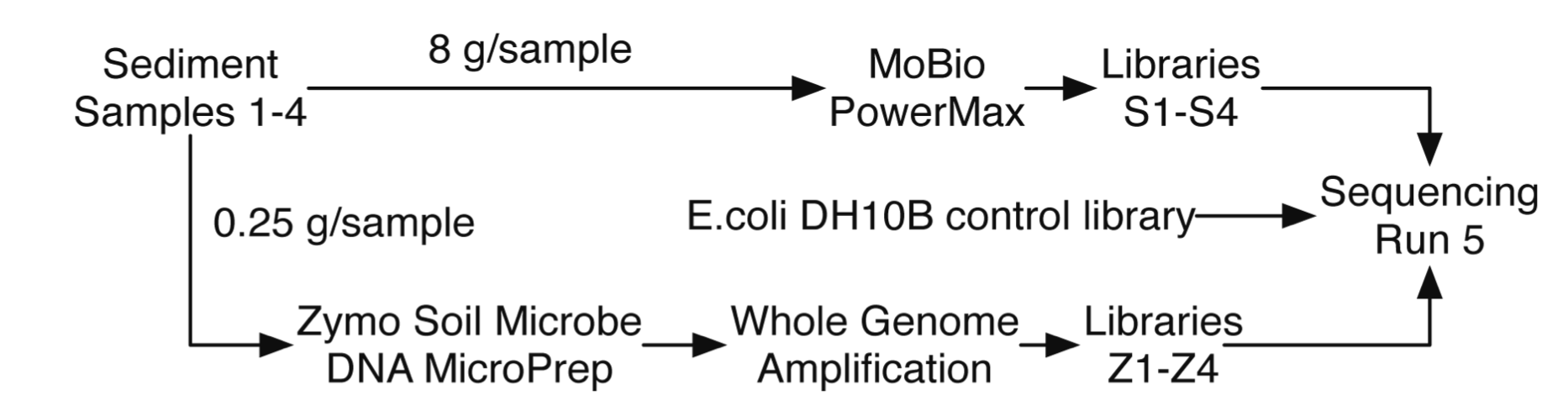

Supplement: Supplementary file 3 [file Image3.TIFF]

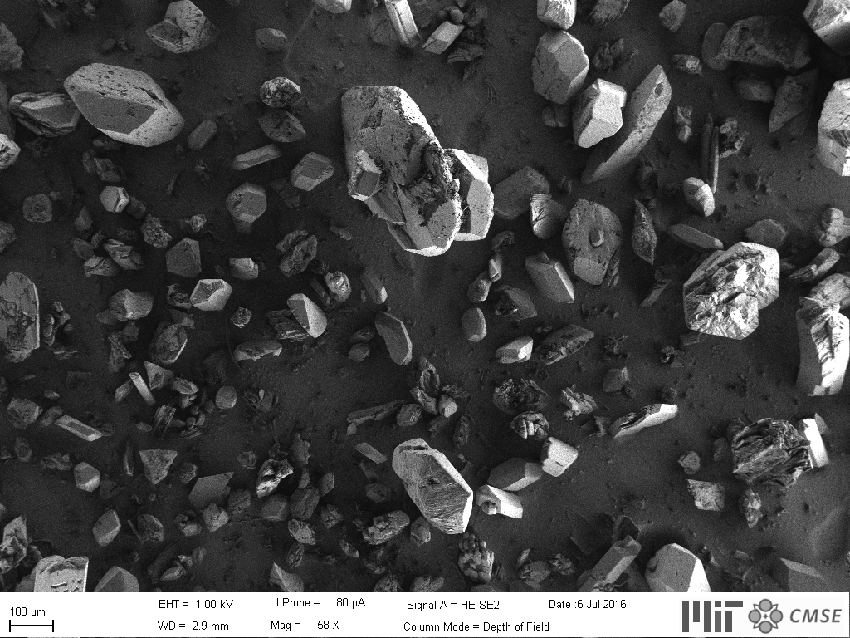

Supplement: Supplementary file 4 [file Image4.TIF]

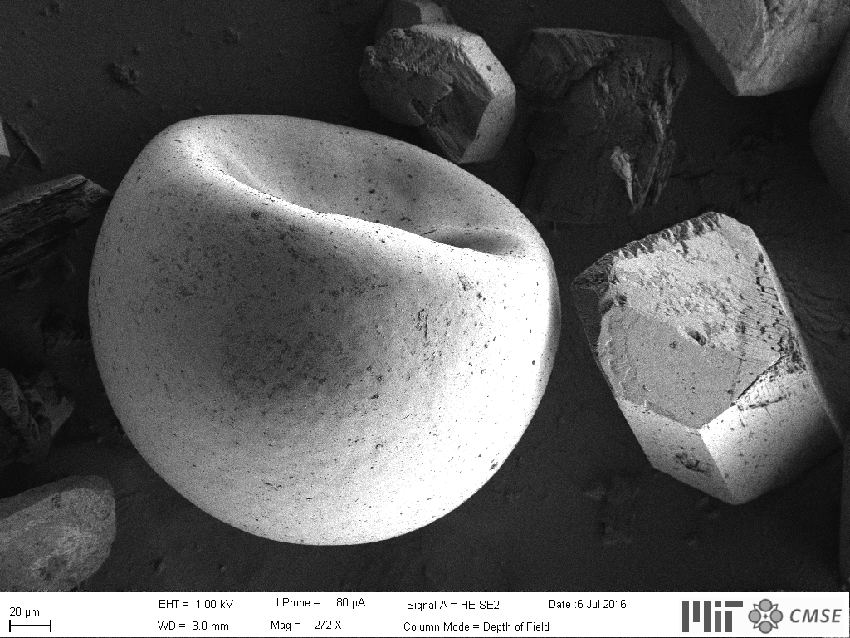

Supplement: Supplementary file 5 [file Image5.TIF]

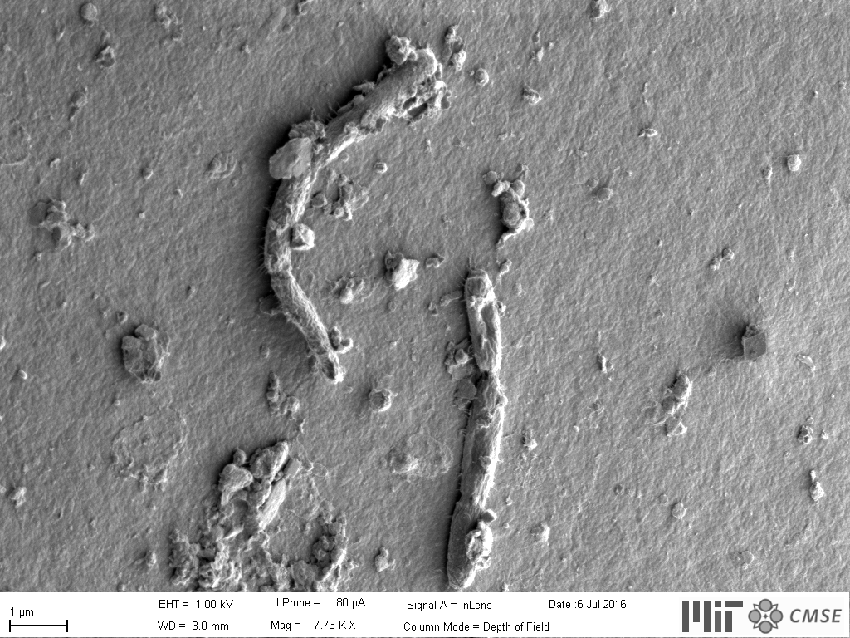

Supplement: Supplementary file 6 [file Image6.TIF]

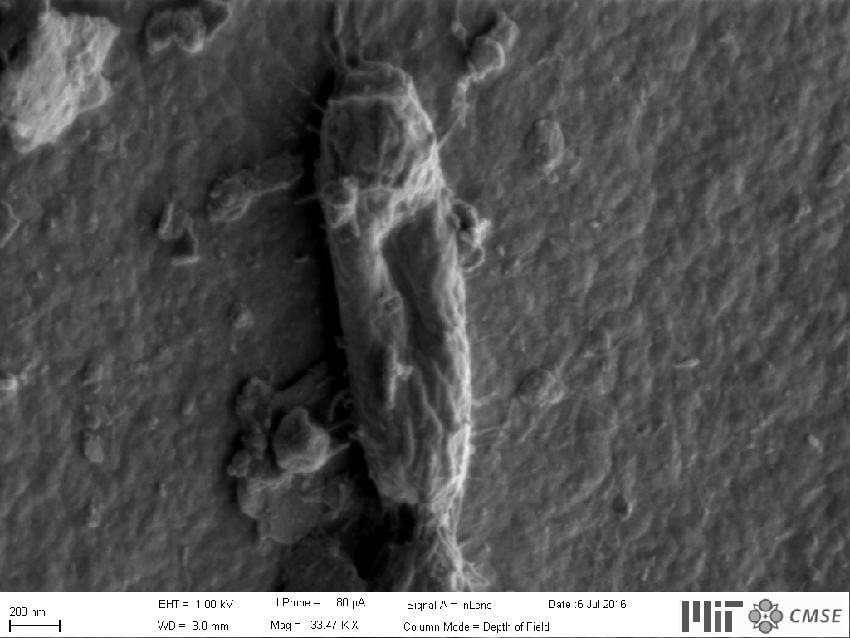

Supplement: Supplementary file 7 [file Image7.TIF]

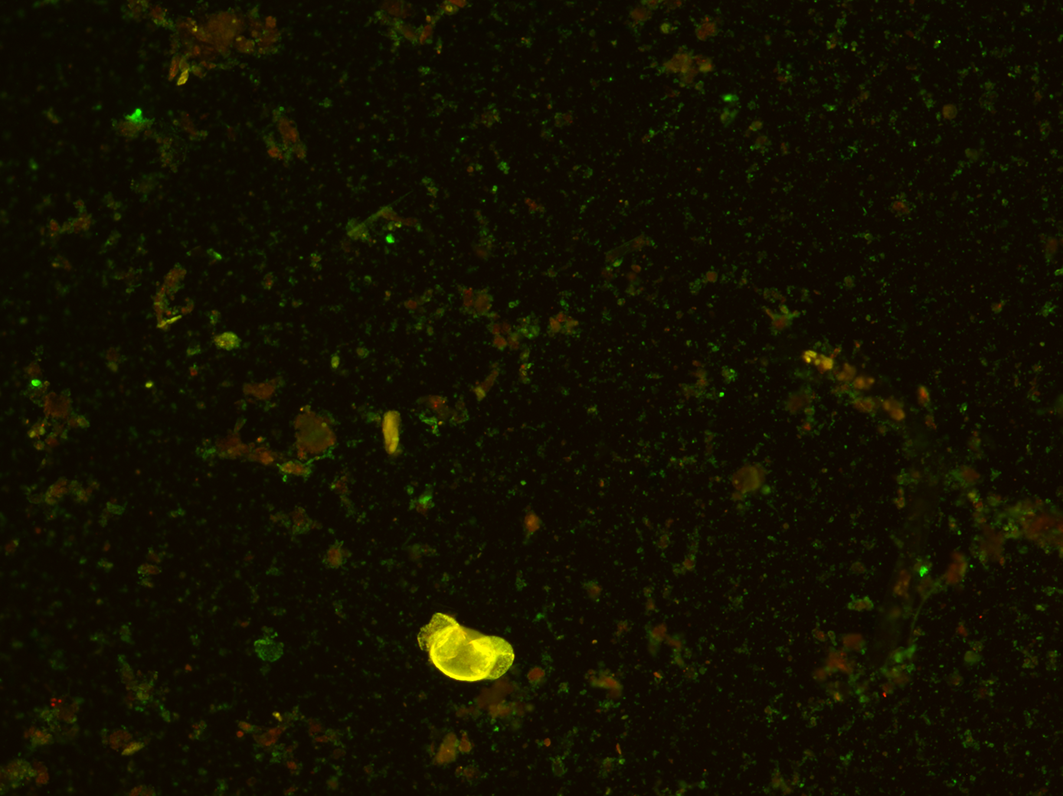

Supplement: Supplementary file 8 [file Image8.TIF]
