# Supplementary material for: Microbial Diversity in a Hypersaline Sulfate Lake: A Terrestrial Analog of Ancient Mars
Source: Front Microbiol. 2017 Sep 26;8:1819. doi: 10.3389/fmicb.2017.01819 (PMC5623196; doi:10.3389/fmicb.2017.01819)
Supplement: Supplementary file 9 [file Table1.DOCX]

**Table S1. Sequencing Summary by Sample**

| Method | Sample (Library) | Runs | Total Bases (Mbp) | ≥Q20 Bases (Mbp) | Reads (10^3^) | Mean Read Length (bp) |
| --- | --- | --- | --- | --- | --- | --- |
| MoBio PowerMax | S1 | 5 | 31 | 22 | 192 | 161 |
| MoBio PowerMax | S2 | 5 | 26 | 18 | 165 | 155 |
| MoBio PowerMax | S3 | 5 | 20 | 14 | 114 | 176 |
| MoBio PowerMax | S4 | 5 | 19 | 14 | 112 | 169 |
| Zymo MicroPrep + WGA | Z1 | 5 | 32 | 23 | 214 | 150 |
| Zymo MicroPrep + WGA | Z2 | 5 | 27 | 19 | 184 | 147 |
| Zymo MicroPrep + WGA | Z3 | 5 | 23 | 16 | 137 | 166 |
| Zymo MicroPrep + WGA | Z4 | 5 | 37 | 26 | 236 | 158 |
| (E. coli DH10B Control) | C | 5 | 125 | 94 | 642 | 195 |

*Run on Ion Torrent chip 316, with a loading density of 79%. Signal analysis and base calling by TorrentServer 3.2.1
