# Supplementary material for: Microbial Diversity in a Hypersaline Sulfate Lake: A Terrestrial Analog of Ancient Mars
Source: Front Microbiol. 2017 Sep 26;8:1819. doi: 10.3389/fmicb.2017.01819 (PMC5623196; doi:10.3389/fmicb.2017.01819)
Supplement: Supplementary file 10 [file Table2.DOCX]

**Table S2. MG-RAST Searches To Identify Comparison Metagenomes**

| Search Criteria |
| --- |
| project=”salt” or ”hypersaline” or name=”hypersaline” |
| feature=”saline lake” or “alkaline salt lake” or ”saline evaporation pond” or “saline lake sediment” |
| biome=”marine salt marsh biome” or “high osmolarity habitat” or “extreme habitat; hypersaline” |
| project=”Antarctica” or name=”Antarctica” or feature=”ice mass” |
| biome=”air” |
| project=”Global Ocean” |
