# Supplementary material for: Microbial Diversity in a Hypersaline Sulfate Lake: A Terrestrial Analog of Ancient Mars
Source: Front Microbiol. 2017 Sep 26;8:1819. doi: 10.3389/fmicb.2017.01819 (PMC5623196; doi:10.3389/fmicb.2017.01819)
Supplement: Supplementary file 11 [file Table3.DOCX]

**Table S3. MG-RAST Metagenomic Datasets for Comparison**

| **Label** | **Site** | **Description** | **IDs** |
| --- | --- | --- | --- |
| Hypersaline | Chula Vista | Marine: Saline evaporation pond | 4440416.3, 4440425.3, 4440434.3, 4440435.3, 4440426.3, 4440324.3, 4440437.3, 4440090.3, 4440438.3, 4440433.3, 4440429.3, 4440430.3, 4440419.3 |
|  | Cuatro Cienegas | Alkaline salt lake | 4441363.3, 4442466.3 |
|  | GeoBio2012* | Sulfur spring biofilm | 4546047.3 |
|  | GeoBio2013* | Hypersaline water: Great salt lake | 4546082.3, 4546083.3, 4546084.3, 4546085.3, 4546086.3, 4546087.3, 4546088.3 |
|  | Guerrero Negro* | Saline evaporation pond, microbial mat | 4533376.3, 4533377.3, 4533378.3, 4533379.3, 4533380.3, 4533381.3, 4533382.3, 4533383.3 |
|  | LH_CGN: Axel Heiberg, Nunavut | High osmolarity sulfur spring; sulphate-reducing sediment | 4478244.3 |
|  | Mar Menor | Mediterranean Lagoon | 4516291.3, 4516292.3, 4516293.3 |
|  | Marine Salt Marsh | Wood Neck Beach, Falmouth | 4517592.3, 4516362.3 |
|  | NaCl Brine, Santa Pola | Crystallizer pond CR30, "Bras del Port" Saltern | 4441050.3 |
|  | Salton Sea | Hypersaline water, California | 4440329.3 |
|  | Sludge, Aalborg, Denmark | Saline evaporation pond, activated sludge | 4487554.3 |
|  | Yucatan Groundwater | Saline/sulfidic Cenotes, Mexico | 4536379.3, 4536390.3 |
| Spotted Lake | Spotted Lake | Anoxic, sulfidic sediment samples from several pools and sample prep methods | 4562177.3, 4562178.3, 4562179.3, 4562180.3, 4562181.3, 4562182.3, 4562183.3, 4562184.3, 4562185.3, 4562186.3, 4562187.3, 4562188.3 |
| Ocean | Global Ocean Sampling Expedition | Sargasso Sea and Punta Cormorant, Hypersaline Lagoon | 4441570.3, 4441571.3, 4441574.3, 4441576.3, 4441599.3 |
| Polar | Antarctica | Small lake Biome | 4487042.3 |
|  | Antarctica Aquatic | Ace Lake (meromictic, sulfidic), Organic Lake, Marine Environments | 4443683.3, 4443680.3, 4443679.3, 4443681.3, 4443682.3, 4443684.3, 4443685.3, 4443686.3, 4443687.3 |
|  | Arctic Ice | Marginal sea biome: Barrow, Alaska | 4537102.3, 4537103.3, 4537104.3, 4537105.3 |
| Air | Beijing Smog+ | Beijing’s PM2.5 and PM10 Pollutants Time Series | 4516637.3, 4516911.3, 4516455.3, 4516910.3, 4516594.3, 4516459.3, 4516803.3, 4516651.3, 4516952.3, 4516802.3, 4516402.3, 4517064.3, 4516403.3, 4516366.3 |
|  | Storm Peak* | Air samples | 4456075.3, 4456076.3, 4456077.3, 4456078.3, 4456079.3, 4456081.3, 4456082.3, 4456083.3, 4456084.3 |
|  | Indoor Air* | Shopping Center, Singapore | 4465825.3, 4465946.3 |

* Amplicon-based dataset used only in abundance analysis. ^+^Excluded from functional analysis due to large size of dataset.
