# Supplementary material for: Microbial Diversity in a Hypersaline Sulfate Lake: A Terrestrial Analog of Ancient Mars
Source: Front Microbiol. 2017 Sep 26;8:1819. doi: 10.3389/fmicb.2017.01819 (PMC5623196; doi:10.3389/fmicb.2017.01819)
Supplement: Supplementary file 12 [file DataSheet1.docx]

Supporting Information

Pontefract et al.

# Supporting References

1. Lindemann SR*, et al.* (2013) The epsomitic phototrophic microbial mat of Hot Lake, Washington: community structural responses to seasonal cycling. *Frontiers in microbiology* 4:323.

2. McKay E (1935) Salt Tolerance of Ruppia maritima in Lakes of High Magnesium Sulphate Content. *Plant Physiology* 10(3):425-446.

# Supporting Figure Legends

**Fig. S1. Site and Sample Collection.** Spotted Lake (left panel; GPS: 570 m elevation N 49.078018˚ W 119.567502˚) with samples collected by V. K. Walker (right panel) on October 24, 2010.

**Fig. S2. Modern and historical geochemistry of Hot Lake and Spotted Lake.** Water balance drives seasonal changes in geochemistry of major ions in Hot Lake (Lindemann et al. 2013) and Spotted Lake. Historical data for Hot Lake (McKay 1935) reveals consistent but variable high ion concentrations, and is consistent with present day measurements.

**Fig. S3. Metagenomic sequencing overview.** Traditional and “low-input” approaches. See Methods for details.

**Fig. S4.** Original SEM micrograph for plate A, Figure 2.

**Fig. S5.** Original SEM micrograph for plate B, Figure 2.

**Fig. S6.** Original SEM micrograph for plate C, Figure 2.

**Fig. S7.** Original SEM micrograph for insert in plate C, Figure 2.

**Fig. S8.** Original Live/Dead baclight image for plate D, Figure 2.

**Data Deposit:**

Data deposition: Sequencing data was deposited with NCBI under BioProject PRJNA245804 and SRA entry SRP041543: SRR1266728-SRR1266731 (S1-S4), SRR1266732-SRR1266735 (Z1-Z4), and SRR1266736-SRR1266739 (D2 Runs 1-4). Corresponding MG-RAST metagenome IDs: 4562181.3 (S1), 4562182.3 (S2), 4562183.3 (S3), 4562184.3 (S4), 4562185.3 (Z1), 4562186.3 (Z2), 4562187.3 (Z3), 4562188.3 (Z4), 4562177.3 (D2 Run 1), 4562178.3 (D2 Run 2), 4562179.3 (D2 Run 3), and 4562180.3 (D2 Run 4).

**PCA Analysis:**

A separate PCA analysis was done for each taxonomic level from domain to species by summing the abundance for each unique key within each metagenome. The abundance matrix was normalized to unity sum across rows to account for differences in abundance due purely to the size of datasets.

Next, PCA was performed using the *pca()* function in MATLAB (The Mathworks, Natick, MA) using the default options (centering of data by subtracting column means, svd algorithm). The taxonomic signature associated with each $PC=\left[ \begin{matrix} w_{1} & \ldots& w_{n} \end{matrix} \right]$ was determined as the unique taxonomic key with the highest magnitude weighting ($w_{max}$) for a given PC, and the figure of merit was calculated as:

$$FoM=\sqrt{\frac{\left( w_{max} \right)^{2}}{\sum_{i=1}^{n} \left( w_{i} \right)^{2}}}$$

This figure of merit ranges from 1/n (approaching 0 as n→∞) for no association between a PC score and a taxonomic key (all n $w_{i}$ are equal) and 1 when changes in PC score produce only changes in a single taxonomic key and all but a single $w_{i}$ are zero.

Similarly, PCA analysis and FoM calculation was performed using functional abundance data generated via SEED Subsystem classification with the MG-RAST default settings (max e-value 10^-5^, min identity cutoff 60%, min alignment length cutoff 15 bp).
